# Snapshot imaging of ultrashort electron bunches

**DOI:** 10.1038/s41377-024-01489-z

**Published:** 2024-06-12

**Authors:** Andreas Döpp

**Affiliations:** 1 Ludwig-Maximilian-Universität München, Am Coulombwall 1, 85748 Garching, Germany; 2 Max Planck Institut für Quantenoptik, Hans-Kopfermann-Strasse 1, Garching, 85748 Germany

**Keywords:** Optical physics, Laser-produced plasmas

## Abstract

New measurements combine spatial and temporal information from optical transition radiation to estimate the three-dimensional structure of electron bunches from a laser wakefield accelerator.

Laser-plasma accelerators (LPAs) driven by high-power lasers can generate electron bunches with femtosecond durations and kilo-ampere currents in just a few millimeters, rivaling state-of-the-art conventional accelerators in a much more compact setup^1,2^. While research into the physics of laser wakefield accelerators continues to advance^3–5^, future high-impact applications—such as ultrafast electron diffraction and X-ray generation—demand exceptional quality from the generated electron beams^6^. Knowledge about the spatiotemporal structure of the accelerated electrons is therefore crucial. However, measuring such ultra-short bunches is highly challenging and has been mostly limited to either the transverse or longitudinal profiles^7–12^.

Now, writing in *Light: Science & Applications*, Kai Huang and co-workers from the Kansai Institute for Photon Science, Osaka University, and RIKEN in Japan have succeeded in measuring the three-dimensional (3D) density distribution of a laser-wakefield accelerated electron pulse with femtosecond temporal resolution and micrometer spatial resolution^13^. This single-shot measurement was enabled by combining imaging of optical transition radiation (OTR) - which encodes the transverse profile—with electro-optic (EO) sampling of the OTR—which provides information about the temporal structure (Fig. 1).

**Fig. 1:**
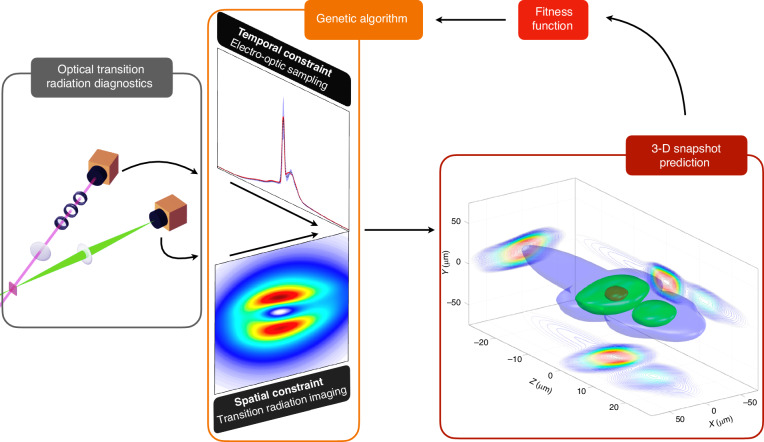
**Schematic view of the snapshot retrieval method by Huang and colleagues**. The method relies on the simultaneous measurement of optical transition radiation (OTR) through imaging and electro-optic sampling. Combined, these two diagnostics constrain the temporal and spatial structure of the electron bunch that generated the OTR. The 3D structure is then estimated by optimizing a multi-Gaussian distribution through an evolutionary search algorithm that matches the prediction from the model to the experimental data. Images adapted from [ref. ^13^]

To perform the measurement, a stainless-steel foil was placed 7 cm behind the exit of the LPA to generate optical transition radiation. The OTR signal was then imaged both onto a camera for transverse profiling and onto a thin gallium phosphide (GaP) crystal for EO sampling^14^. Within the GaP crystal, the temporal information of the electron bunch is imprinted onto the transverse profile of a probe laser beam. Assuming the electron distribution within the bunch as a multi-Gaussian distribution, the 3D structure was constrained down to the few-micrometer and few-femtosecond level using a genetic algorithm in conjunction with a detailed numerical model of the diagnostic^15^. The authors estimate that the electron bunches had a duration of just a few femtoseconds with a peak current exceeding 1 kA. The transverse size was measured to be less than 30 µm (root mean square) with an estimated peak 3D density of 9 × 10^21^ m^−3^. Knowledge of this density is crucial for many applications and, in the case of the authors’ research, is motivated by the goal to build a compact, laser-plasma driven free-electron laser (FEL). Knowledge of the electron density and the related Pierce parameter is essential to estimate the gain length of the FEL.

The approach used by Huang et al. serves as a compelling example in a greater trend towards novel diagnostics that capture the spatiotemporal structure of intense beams—particles and photons alike. Due to the inherent limitations of 2D detectors to capture 3D structures, snapshot approaches inherently rely on data-driven techniques^16^. Measurements resemble tomographic reconstruction as they capture the beam under scrutiny at different “angles”; by combining multiple diagnostics that each provide a partial constraint, the properties of the beam can be inferred. The study by Huang et al. serves as an encouraging proof-of-principle and cross-fertilization with concepts employed in computed tomography or laser diagnostics may give room for rapid future progress, e.g., by borrowing techniques from compressed sensing^17,18^.

The development of improved diagnostics goes hand-in-hand with advances in the fundamental understanding and control of laser-plasma accelerators. For instance, femtosecond electron microscopy as recently introduced to study relativistic electron bunches^19^ can also serve as formidable diagnostic laser-plasma wakefield dynamics^20^. Similarly, combining OTR imaging and electro-optic sampling with spectroscopic measurements of coherent transition radiation in the THz regime may help better understand the complex physics of beam-driven wakefield accelerators^3,4^ or to diagnose “exotic” electron beams^21^. Machine learning techniques in particular may provide a powerful framework for integrating these diverse measurements and extracting meaningful correlations. The complexity of laser-plasma accelerators, with their many coupled parameters, makes them well-suited to such data-driven analysis approaches. The multi-modal reconstruction put forward by Huang and colleagues not only demonstrates a new capability for capturing 3D electron bunch structure, but also points the way towards future innovations in data-driven discovery and optimization for laser-plasma accelerators.
